# Piloting the Virtual PLAYshop Program: A Parent-Focused Physical Literacy Intervention for Early Childhood

**DOI:** 10.3390/children10040720

**Published:** 2023-04-13

**Authors:** Yeongho Hwang, Madison Boyd, Patti-Jean Naylor, Ryan E. Rhodes, Sam Liu, Ramiah Moldenhauer, Joshua Li, Chris Wright, E. Jean Buckler, Valerie Carson

**Affiliations:** 1 Faculty of Kinesiology, Sport, and Recreation, University of Alberta, Edmonton, AB T6G 2H9, Canada; 2 School of Exercise Science, Physical and Health Education, University of Victoria, Victoria, BC V8W 2Y2, Canada

**Keywords:** physical literacy, physical activity, fundamental movement skills, parent, early childhood, preschool, virtual intervention, COVID-19

## Abstract

The PLAYshop program is a parent-focused physical literacy intervention for early childhood. This single-group mixed-methods pilot study aimed to explore the feasibility of virtually delivering and assessing the PLAYshop program. The virtual PLAYshop program included a virtual workshop, resources/basic equipment, and two booster emails (3-week and 6-week follow-up). Data on 34 preschool-aged children (3–5 years) and their parents from Edmonton and Victoria, Canada, were collected via an online questionnaire, virtual assessment session, and interview at single or multiple time points (baseline, post-workshop, 2-month follow-up). Intraclass correlation coefficients (ICCs), paired *t*-tests, repeated measures ANOVAs, and thematic analyses were conducted. Regarding feasibility, most parents (≥94%) were satisfied/extremely satisfied with the virtual workshop and planned to continue physical literacy activities post-workshop. The virtual assessment protocol for children’s fundamental movement skills (FMS; overhand throw, underhand throw, horizontal jump, hop, one-leg balance) was feasible, with high completion rates (>90%) and reliable scoring (ICC = 0.79–0.99). For positive changes in potential outcomes, a medium effect size was observed for children’s hopping skills (*d* = 0.54), and large effect sizes were observed for several parental outcomes (partial *η*^2^ = 0.20–0.54). The findings support the feasibility and potential positive outcomes of the virtual PLAYshop program. A larger randomized controlled efficacy trial is recommended.

## 1. Introduction

Evidence consistently suggests that physical activity is positively associated with physical, psychosocial, and cognitive health in pediatric age groups [1,2]. However, studies analyzing nationally representative samples of Canadians indicated that only 61.8% of children aged 3–4 [3] and 39% of children aged 5–17 years met national physical activity recommendations for optimal health [4]. On top of this, COVID-19-related restrictions have been found to unintentionally reduce physical activity in children and youth worldwide [5,6]. Given that physical inactivity is considered a public health crisis [7], it is necessary to explore ways to promote physical activity.

Physical literacy is attracting attention as a concept for ameliorating insufficient physical activity across the lifespan [8,9]. Physical literacy is defined by the International Physical Literacy Association (IPLA) as “the motivation, confidence, physical competence, knowledge and understanding to value and take responsibility for engagement in physical activities for life” [10]. This IPLA definition includes affective (e.g., motivation and confidence); physical capability (e.g., physical competence and fundamental movement skills (FMS)); and cognitive (e.g., knowledge and understanding of activities) components [11]. Notably, previous research suggests that the development of these core components of physical literacy in the preschool years (3–5 years) may be a promising approach for promoting physical activity participation and subsequent health benefits throughout the life course [12].

The preschool years are a critical period of physical literacy development [13]; however, how best to intervene to support the development of physical literacy in preschool-aged children remains unclear. Previous evidence has shown that meaningful parental involvement plays a crucial role in shaping physicalactivity-related behaviors in children [14,15,16,17]. This is especially true for preschool-aged children considering they are dependent on their parents because of their limited autonomy. Based on the evidence, the critical role of parents in supporting the development of their children’s physical literacy has been emphasized [13]. However, to date, few studies have investigated parent-focused physical literacy interventions for preschool-aged children. 

In a systematic review published in 2022 [18], it was reported that a small number of physical literacy interventions (n = 4) were conducted specifically targeting preschool-aged children [19,20,21,22]. All four of these interventions were conducted in childcare settings [19,20,21,22]. Therefore, understanding how parents can best support their preschool-aged children’s physical literacy development in home and neighborhood settings remains unclear. Additionally, the four physical literacy interventions were lengthy (e.g., 6–8 months) and classified within the review as theory-inspired rather than theory-based [18]. The PLAYshop program, a brief, theory-based, parent-focused physical literacy intervention, was developed to address current evidence gaps [23,24]. Previous work has found the PLAYshop program to be feasible and efficacious [23,24]. Furthermore, Carl et al.’s review (2022) classified the PLAYshop program as theory-based because of the extensive links between the intervention content and core physical literacy components [18].

During the COVID-19 pandemic, public health restrictions and concerns over contracting the virus made it difficult to offer and access face-to-face, parent-focused physical literacy interventions. Given the pandemic, a virtually delivered version of the PLAYshop program was one viable option to support parents and their preschool-aged children. This aligns with other pre-COVID-19 interventions delivered virtually in Canada aiming to promote physical activity among preschool-aged children by targeting childcare providers [25,26]. In addition to converting the PLAYshop program into a virtual format, a virtual protocol to assess preschool-aged children’s FMS was also developed. FMS are commonly assessed to measure the physical capability component of children’s physical literacy [11]. Our new virtual protocol focused on FMS because existing valid and reliable tools to assess FMS in preschool-aged children, such as the Test of Gross Motor Development-Third Edition (TGMD-3) [27], are typically conducted through in-person direct observation. We did not develop a new virtual protocol for other physical literacy components, such as the affective and cognitive components, because direct observation and, therefore, adaptation to a virtual protocol are not required.

When adapting an existing intervention to a different format, conducting a small, inexpensive pilot study is a valuable step to inform whether a larger randomized controlled efficacy trial is needed in the future [28]. Therefore, a pilot study, using a single-group design, was conducted to explore the feasibility and some potential outcomes of a virtually delivered PLAYshop program. Specifically, the primary objectives of this study were to (1) explore the parental experiences of the virtual PLAYshop program; and (2) examine the feasibility of a virtual assessment protocol for preschool-aged children’s FMS. The secondary objectives were to examine the potential outcomes of participating in the virtual PLAYshop program. Specifically, this study examined if participating in the virtual PLAYshop program: (1) improved preschool-aged children’s FMS; (2) improved parental capability, opportunity, and motivation to support their children’s physical literacy development; and (3) improved parental physical activity modeling and parent-child co-participation in physical activity. Note that it was not an objective of this trial to comprehensively assess children’s physical literacy.

## 2. Methods

### 2.1. Study Design

A single-group mixed-methods design was employed for this pilot study. This study is reported according to the applicable items from the Transparent Reporting of Evaluations with Nonrandomized Designs (TREND) statement for nonrandomized evaluations of behavioral and public health interventions [29] and an extension to the Consolidated Standards of Reporting Trials (CONSORT) statement for randomized pilot and feasibility trials [30,31].

### 2.2. Participants

Families were recruited through online advertising (i.e., paid Facebook advertising) from June to August 2021. To be eligible, families had to have a preschool-aged child (3–5 years) and live in or around two Canadian cities: Edmonton, Alberta or Victoria, British Columbia. Since the virtual workshop, a core component of the virtual PLAYshop program, and the educational materials were in English for the pilot, families with parents who could not comfortably speak or read English were excluded. Additionally, families without access to a smartphone, tablet, or laptop with a camera and microphone were excluded.

A total of 40 families completed screening interviews for study eligibility. Three families were ineligible because the child was not 3 to 5 years old, and another three eligible families were not available for the workshop dates. Accordingly, 34 families consented to participate in the study at baseline. Human research ethics approval was obtained from the University of Alberta (Ethics Project #: 00093764; date of approval: 12 September 2019) and the University of Victoria (Ethics Project #: 16-444; date of approval: 30 July 2019). Participating parents provided written informed consent via REDCap, an electronic data capture tool [32]. Additionally, verbal consent was obtained from parents prior to any video or audio recording. As the purpose of this pilot study was to evaluate the feasibility and potential outcomes of the virtual PLAYshop program prior to a randomized controlled trial, sample size calculations were not performed [30].

### 2.3. Intervention (Virtual PLAYshop Program)

**Figure 1:**
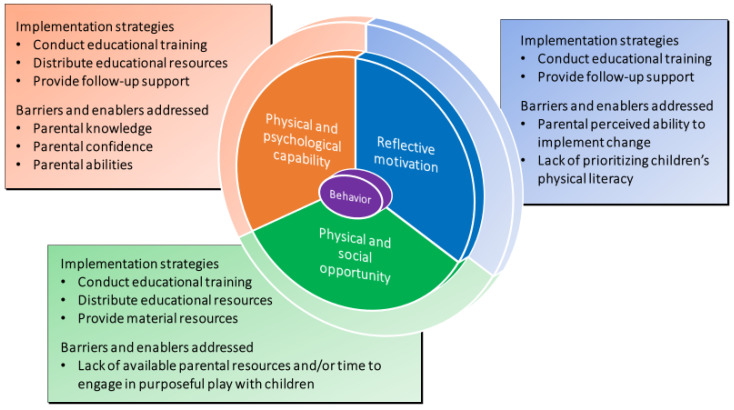
Implementation strategies of the virtual PLAYshop program and the barriers and enablers that were addressed and mapped to the COM-B model.

The specific aims, development process, and logical foundation of the original in-person PLAYshop program have been previously reported [23]. Briefly, the overall aim of the PLAYshop program was to build parental capability, opportunity, and motivation to support their children’s physical literacy development and physical activity acquisition through purposeful play. The PLAYshop program, designed as a concise and scalable physical literacy intervention, aimed to alleviate common parental barriers such as time, cost, and opportunity [33] and was informed by evidence-based behavior change techniques corresponding to the Capability-Opportunity-Motivation = Behavior (COM-B) model [34]. The COM-B model serves as the inner circle of the Behavior Change Wheel, a well-established meta-theory commonly employed in the field of physical activity research [35,36,37]. Lane et al. [24] provided detailed information regarding the implementation strategies, intervention functions, barriers and enablers addressed, behavior change techniques employed, and descriptions of the PLAYshop program. Figure 1, adapted from Lane et al. [24], shows the implementation strategies of the virtual PLAYshop program and the barriers and enablers that were addressed and mapped to the COM-B model.

The original in-person PLAYshop program was converted to a virtual format and included four core components or implementation strategies. First, a 75 min, virtual, synchronous, free parent workshop was delivered, including interactive activities and educational messages embedded with physical literacy concepts. Second, educational resources (i.e., hardcopy handouts) were distributed. Third, material resources (i.e., a bag of inexpensive active play equipment (e.g., a small ball, beanbag, scarf, and balloons)) were provided. Fourth, follow-up support after the virtual workshop (i.e., two post-workshop booster emails (3-week and 6-week follow-up)) was provided. 

### 2.4. Procedures

**Figure 2:**
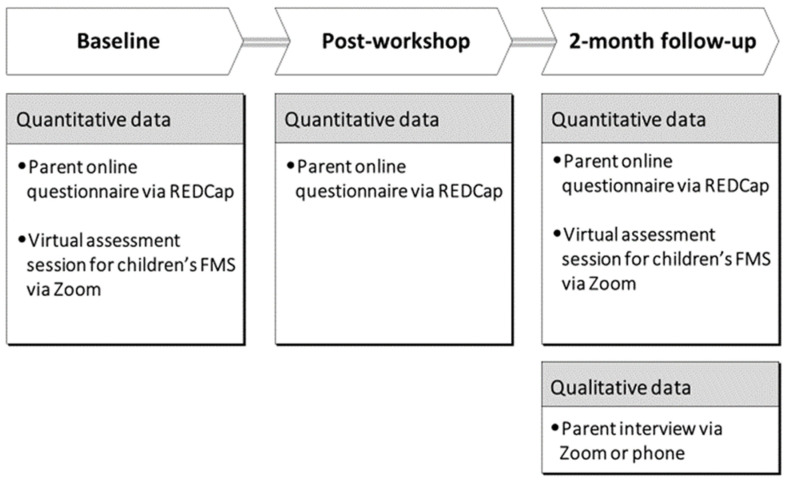
Overall procedures for data collection.

The overall procedures for data collection are outlined in Figure 2. Data on study outcomes and participant characteristics from one designated parent and their child were obtained. The designated parent was the person who was identified as spending the most time with their child during play activities. The research team collected quantitative data at baseline, post-workshop, and 2-month follow-up and qualitative data at 2-month follow-up. 

At baseline, parents filled out an online questionnaire via REDCap and attended a virtual assessment session for children’s FMS via Zoom with their child led by a trained research staff member. At the session, parents and children watched a pre-recorded demonstration video before each of the FMS assessed (i.e., overhand throw, underhand throw, horizontal jump, hop, one-leg balance). Then, parents made preparations for their children’s FMS trials (e.g., creating a starting line based on the staff’s instructions). Children’s skills were filmed via parents’ smartphones, tablets, or laptops, while the virtual assessment session was recorded for later scoring. Each FMS assessment is described in detail in the data collection section. Within ten days after the baseline data collection, parents and their children (optional) participated in the virtual workshop at the selected date and time, and the research team mailed the participants a bag containing inexpensive active play equipment (e.g., ball, bean bag, balloon) and handouts prior to their scheduled virtual workshop. Post-workshop, parents completed a second online questionnaire via REDCap. At 3-week and 6-week follow-ups, the research team sent parents a booster email with key workshop messages, encouragement for parents, and new activity ideas. At 2-month follow-up, parents filled out a third online questionnaire via REDCap, and once again virtual assessment sessions for children’s FMS were recorded. Additionally, brief semi-structured interviews were conducted with the designated parent immediately after the virtual assessment sessions for children’s FMS were recorded. These interviews were audio-recorded.

### 2.5. Data Collection

#### 2.5.1. Parental Experiences of the Virtual PLAYshop Program (Primary Objective 1)

Consistent with previous research on the original in-person PLAYshop program [23], parents responded to four items of usefulness, satisfaction, and novelty related to the virtual workshop on the second parent online questionnaire immediately after the virtual workshop: The usefulness of the virtual workshop was measured on a 5-point Likert scale from 1 (not useful) to 3 (somewhat useful) to 5 (extremely useful). Satisfaction with the virtual workshop content and delivery was also measured on a 5-point Likert scale from 1 (not satisfied) to 3 (somewhat satisfied) to 5 (extremely satisfied). Finally, the novelty of the virtual workshop content was measured on a 5-point Likert scale from 1 (not new) to 3 (somewhat new) to 5 (very new). 

Qualitative data were collected through the parent interview at 2-month follow-up. The parent interview consisted of two main questions with follow-up prompts when needed. First, parents were asked about their children’s participation in activities that build physical literacy since the virtual workshop, how the activities went, and the facilitators and barriers to the activities. Second, parents were asked about their likelihood of continuing activities that build their children’s physical literacy in the future. Parents were also given the opportunity to add any additional comments about their experiences of the virtual PLAYshop program.

#### 2.5.2. Virtual Assessment Protocol for Children’s FMS (Primary Objective 2)

Children’s FMS data were collected via virtual assessment sessions at baseline and 2-month follow-up. The feasibility of the virtual assessment protocol was examined by calculating the completion rates and the inter-rater reliability of the FMS scores at baseline and 2-month follow-up. Research staff were trained to establish inter-rater reliability through pilot videos. Then, one staff member scored all of the videos, and another staff member scored 20% of the same videos according to pre-established performance criteria [27,38].

#### 2.5.3. Children’s FMS (Secondary Objective 1)

Five FMS were assessed virtually at baseline and 2-month follow-up. These skills included two manipulative/ball skills (i.e., overhand throw, underhand throw); two locomotor skills (i.e., horizontal jump, hop) from the TGMD-3 [27]; and one balance/stability skill (i.e., one-leg balance) from the field-based FITness testing in PREschool children (PREFIT battery) [38]. These five skills were selected because they required minimal space and equipment and were consistent with the content of the virtual workshop. Parents were asked not to provide children with any verbal or physical cues (e.g., demonstrating) as to how to perform the skills. Children completed one practice trial and then two test trials for the manipulative/ball and locomotor skills. For one-leg balance, children completed two practice trials (one per leg) and then two test trials (one per leg). The overhand throw, underhand throw, horizontal jump, and hop were scored according to the standard performance criteria, and their scores ranged from 0 to 8 [27]. One-leg balance was assessed by timing the right and left foot test trials. The final balance score was the best (longest) time of the two. The time range for the one-leg balance was unlimited, and the time was recorded to two decimal places.

#### 2.5.4. Parental Capability, Opportunity, and Motivation (Secondary Objective 2)

Items related to parental capability (i.e., knowledge); opportunity (i.e., perceived availability of resources, perceived barriers); and motivation (i.e., confidence, beliefs, outcome expectations) to support their children’s physical literacy development were measured at all time points (i.e., baseline, post-workshop, and 2-month follow-up). Parental knowledge was measured with nine items (e.g., manipulative/ball skills (catching, hitting, striking, kicking, throwing, etc.) and creating a home environment that encourages active play) based on the core components of physical literacy [13]. The response categories for these items were measured on a 5-point Likert scale from 1 (no knowledge) to 3 (some knowledge) to 5 (a lot of knowledge). The scores of the nine items were summed, ranging from 0 to 45, with higher scores representing more parental knowledge. The Cronbach’s alpha values were 0.947 (baseline), 0.915 (post-workshop), and 0.912 (2-month follow-up). 

The parental perceived availability of resources was measured with a single item, consistent with the previous study on the original in-person PLAYshop program [24]. The response categories for this item were measured on a 5-point Likert scale from 1 (yes, I have all of the resources I need) to 3 (I have some of the resources I need) to 5 (no, I do not have the resources I need). This item was reverse-coded, with higher scores indicating a higher parental perceived availability of resources. Parental perceived barriers were measured through five items (lack of time, discomfort in letting child play outside, lack of opportunity, transportation problem, high cost) from a previous scale [39]. The response categories for these items were on a 5-point Likert scale from 1 (strongly disagree) to 3 (neutral) to 5 (strongly agree). Higher scores indicated higher parental perceived barriers. The five items were not summed because the internal consistency reliability was low, in particular at baseline. The Cronbach’s alpha values were 0.350 (baseline), 0.628 (post-workshop), and 0.599 (2-month follow-up).

Parental confidence was measured with 11 items (e.g., if I wanted to, I am CONFIDENT in my ability to provide activities to my preschool child that include manipulative/ball skills (e.g., catching, hitting, striking, kicking, throwing); I am CONFIDENT in my ability to create a home environment that encourages active play) based on the core components of physical literacy [13]. The response categories for these items were scored on a 5-point Likert scale from 1 (no confidence) to 3 (some confidence) to 5 (a lot of confidence). The scores of the 11 parental confidence items were summed, ranging from 0 to 55, with higher scores representing more parental confidence. The Cronbach’s alpha values for parental confidence were 0.937 (baseline), 0.947 (post-workshop), and 0.943 (2-month follow-up). Parental beliefs and outcome expectations were measured with four and three items, respectively, from previous scales [39] with minor modifications. The response categories for these items were measured on a 5-point Likert scale from 1 (strongly disagree) to 3 (neutral) to 5 (strongly agree). The scores of the four items for parental beliefs and three items for parental outcome expectations were summed, ranging from 0 to 20 and 0 to 15, respectively. Higher scores represented firmer parental beliefs and higher parental outcome expectations. The Cronbach’s alpha values were 0.857 (baseline), 0.839 (post-workshop), and 0.941 (2-month follow-up) for parental beliefs and 0.645 (baseline), 0.812 (post-workshop), and 0.699 (2-month follow-up) for parental outcome expectations.

#### 2.5.5. Parental Physical Activity Modeling and Parent–Child Co-Participation in Physical Activity (Secondary Objective 3)

Items related to parental physical activity modeling and parent–child co-participation in physical activity were measured at all time points (i.e., baseline, post-workshop, and 2-month follow-up). Parental physical activity modeling was measured with three items from the Activity Support Scale for Multiple Groups (ACTS-MG) [40]. The response categories for these items were scored on a 4-point Likert scale from 1 (strongly disagree) to 4 (strongly agree). The scores of the three items were summed, ranging from 0 to 12, with higher scores indicating higher parental physical activity modeling. The Cronbach’s alpha values were 0.875 (baseline), 0.838 (post-workshop), and 0.872 (2-month follow-up). 

Parent–child co-participation in physical activity was measured with four items from previous research [41]. The response categories for these items were scored on a 5-point scale as follows: 1 (never), 2 (1–2 times per month), 3 (3–4 times per month), 4 (2–3 times per week), and 5 (4 or more times per week). The scores of the four items were summed, ranging from 0 to 20, with higher scores indicating more parent–child co-participation in physical activity. The Cronbach’s alpha values were 0.878 (baseline), 0.843 (post-workshop), and 0.686 (2-month follow-up).

#### 2.5.6. Participant Characteristics

Children’s characteristics included age, sex, race/ethnicity, number of children, nonparental care time, and full-time care. Children’s age (years) was calculated based on reported children’s birth dates and baseline questionnaire completion dates. Children’s sex (assigned at birth) was categorized as either male or female. Children’s race/ethnicity was recorded based on 13 categories (Aboriginal, White, South Asian, Chinese, African, Filipino, Latin American, Arab, Southeast Asian, West Asian, Korean, Japanese, or other) and dichotomized into White or other races/ethnicities. Those who identified as more than one race/ethnicity were also classified into the other races/ethnicities group. The number of children in the household was re-coded as 1, 2, or 3 or more. Nonparental care time (hours per week) was calculated as the sum of hours reported in any of the following five categories: a daycare center, home daycare, another adult in one’s home, another adult outside one’s home, or other. Full-time care was categorized as either yes or no. Full-time care was defined as nonparental care time for 30 h or more per week [42]. 

Parental characteristics included age, sex, the highest level of education, and previous physical literacy-related training. Parental age (years) was calculated based on reported parental birth dates and baseline questionnaire completion dates. Parental sex (assigned at birth) was categorized as either male or female. The parental highest level of education was categorized into above Bachelor’s degree, Bachelor’s degree, or below Bachelor’s degree. Previous physical literacy-related training was categorized as either yes or no. These participant characteristics, which have been used in previous PLAYshop work [23,24], are primarily based on questions developed by Statistics Canada [43].

### 2.6. Data Analyses

#### 2.6.1. Quantitative Analyses

Quantitative data analyses were performed using SPSS Version 28 (IBM Corp., Armonk, NY, USA) and STATA Version 16.1 (Stata Corp., College Station, TX, USA). Where relevant, the patterns of missingness were checked, and statistical test assumptions were evaluated prior to analyses. Descriptive statistics were calculated for participant characteristics. To address primary objective 1, descriptive statistics were conducted for the virtual workshop usefulness, satisfaction, and novelty measures. To address primary objective 2, descriptive statistics were calculated to determine the percentage of participants who completed the FMS assessment at each time point. Additionally, intraclass correlation coefficients (ICCs) were calculated to determine the inter-rater reliability of the FMS scores. To address secondary objective 1, paired samples *t*-tests were conducted to determine the mean changes in children’s FMS scores between baseline and 2-month follow-up. Effect sizes for the mean changes were calculated using Cohen’s *d*. Effect sizes were interpreted as small (absolute value of *d* = 0.20), medium (absolute value of *d* = 0.50), and large (absolute value of *d* = 0.80) [44,45]. To address secondary objectives 2 and 3,repeated measures ANOVAs were conducted to determine the mean changes in the parental capability, opportunity, motivation, physical activity modeling, and parent–child co-participation in physical activity over time (between three time points: baseline, post-workshop, and 2-month follow-up). To determine whether the mean changes found between baseline and post-workshop were maintained at 2-month follow-up, paired samples *t*-tests were performed as follow-ups (baseline vs. post-workshop; baseline vs. 2-month follow-up). Effect sizes for the mean changes were calculated using partial eta squared (*η*^2^). Partial *η*^2^ values of 0.01, 0.06, and 0.14 were interpreted as small, medium, and large effect sizes, respectively [46]. Statistical significance was set at *p* < 0.05.

#### 2.6.2. Qualitative Analyses

Qualitative data analyses were performed using NVivo (QSR International Pty Ltd., Burlington, MA, USA). To address primary objective 1, qualitative data collected from the parent interviews were transcribed using transcription software and checked for accuracy by a research staff member. Interviews were then inductively analyzed using the recommended process for multi-disciplinary health research [47]. One member of the research team coded all interviews first. Then, a second research staff member coded the interviews using codes developed by the first staff member. The two research staff worked collaboratively to chart codes and relationships and develop theme structures. A third researcher confirmed the final categorizations and theme structures. To examine the coverage of codes, sub-themes, and themes specific to parental experiences, frequencies were collected.

## 3. Results

Of the 34 participating families that consented to participate, two did not attend the virtual workshop (too busy: n = 1; unknown: n = 1), and two did not complete the 2-month follow-up components (child injured at daycare center: n = 1; unknown: n = 1). Additionally, one out of 34 children did not participate in the FMS assessment tasks at baseline and 2-month follow-up because the child appeared shy. This resulted in 30 parents providing questionnaire data at all time points (i.e., baseline, post-workshop, and 2-month follow-up) and 29 children with FMS data at baseline and 2-month follow-up. Participant characteristics are presented in Table 1.

Before conducting repeated measures ANOVAs on the variables related to parental capability, opportunity, and motivation (secondary objective 2) and variables related to parental physical activity modeling and parent–child co-participation in physical activity (secondary objective 3), the missingness patterns and statistical test assumptions were checked. In the final analytic sample of 30 parents who completed most questionnaire items at all time points, Little’s [48] missing completely at random (MCAR) tests indicated MCAR patterns (*χ*^2^ = 8.660, *df* = 1092, *p* = 1.000; see Supplementary Table S1). Accordingly, the expectation–maximization (EM) technique was used to estimate missing values. The normality assumption was visually checked, and all variables were assumed to be normally distributed. Mauchly’s test of sphericity showed that all variables met the sphericity assumption, except for the parental perceived barrier of a lack of time and parent–child co-participation in physical activity. For two variables that violated the sphericity assumption, a Greenhouse–Geisser correction was used.

### 3.1. Parental Experiences of the Virtual PLAYshop Program (Primary Objective 1)

Of the 32 parents who completed post-workshop questionnaires, 27 parents found the virtual workshop useful/extremely useful (84.4%), 30 parents were satisfied/extremely satisfied with the virtual workshop content (93.8%), 30 parents were satisfied/extremely satisfied with the virtual workshop delivery (93.8%), and 8 parents felt that the virtual workshop content was new/very new to them (25%). 

Thirty parents participated in the interview at 2-month follow-up. During the semi-structured interviews, parents discussed their children’s participation in physical literacy activities since the virtual workshop, including facilitators and barriers to participation.

#### 3.1.1. Participation

All 30 parents who participated in the interview stated that their child had performed activities from the virtual workshop and/or other physical activities that would support their physical literacy. Parents reported a wide range of activities that involved various FMS (Table 2). Activities that involved manipulative/ball skills were most prevalent, with 27 parents reporting that their child had performed these activities, followed by locomotor skill activities (n = 18) and balance skill activities (n = 6). Other activities such as swimming and biking were also mentioned by many parents (n = 11). Additionally, about half of the parents (n = 16) commented that they had been co-participating in physical literacy activities with their child since the virtual workshop. All parents stated that they planned to continue physical literacy activities in the future with their child.

#### 3.1.2. Facilitators and Barriers

Parents reported several facilitators and barriers to implementing learnings from the virtual PLAYshop program. Sample quotes are presented in Table 3. Themes that emerged through the thematic analyses fell into three levels: parent, child, and the environment. Parent-level themes included physical literacy knowledge; parental personal factors (energy, making physical literacy activities a habit, caring for other children); and the cost of activities. About one third of parents (n = 11) stated that the PLAYshop program had facilitated their ability to implement physical literacy activities by increasing their knowledge in this area. Personal factors such as parental energy (n = 7) and the ability to make physical literacy activities a habit or add these activities to daily routines (n = 6) were cited by parents as both facilitators and barriers. Many parents (n = 7) also mentioned that caring for other children at the same time affected their ability to facilitate physical literacy opportunities. A small number of parents (n = 3) acknowledged that these physical literacy activities could be used as a valuable alternative to paid organized activities, which can be costly.

At the child-level, the themes that emerged were child personal factors (energy, mood, interest) and playful experiences. For example, several parents mentioned that their child’s tendencies, such as energy (n = 8), mood (n = 5), and interest in the activity (n = 13), affected their physical literacy experiences. In some cases, these factors were a facilitator, such as their child often choosing physical activity over other activities and being interested in the new games. In other cases, parents found that their child’s lack of energy after daycare/school or their poor mood was a barrier to physical literacy activities. Despite these barriers, about half of parents (n = 16) commented that the activities they had performed since the workshop felt playful/fun and were enjoyable for their child. 

The environment-level themes included climate, location, minimal equipment, finding time, and the COVID-19 pandemic. Specifically, many parents (n = 18) stated that the climate, including the season and weather, influenced their child’s physical literacy activities. As the virtual PLAYshop program took place between June and August, many parents (n = 14) described that the summer weather had been a facilitator in performing activities. However, parents also acknowledged that the activities demonstrated in the virtual workshop were/would be useful on colder or rainy days when outdoor play is not as enjoyable. Similarly, parents cited that the location of the physical literacy activities, such as outdoors or indoors, affected their experiences. While the majority of parents (n = 25) reported that outdoor spaces facilitated physical literacy activities, several (n = 11) also mentioned being active indoors—though two parents did mention that the space of their homes restricted some of the activities they could perform. Parents also described the equipment required for the workshop activities. Thirteen parents noted that it was helpful that the activities required little equipment and items from around the home could be used rather than expensive equipment. Most parents (n = 18) explained that time was a facilitator or barrier to physical literacy activities. For example, some parents (n = 10) thought the workshop activities were simple and could be performed quickly, to facilitate the frequency of these activities. However, about half of the parents (n = 16) also commented that busy schedules and a lack of time meant they did not perform physical literacy activities as often as they would hope to. Lastly, though only mentioned by four parents, the impact of the COVID-19 pandemic also influenced their child’s physical literacy activities. Of the four parents, one reported that they did not feel the need to perform as many activities at home because their child’s organized activities had returned to normal. In contrast, two parents reported that there were still restrictions on their child’s involvement in organized activities.

### 3.2. Virtual Assessment Protocol for Children’s FMS (Primary Objective 2)

At baseline, the completion rates for children’s FMS were: 90.9% (n = 30/33) for all five FMS, 100% (n = 33/33) for the overhand throw skill, 97% (n = 32/33) for the underhand throw skill, 97% (n = 32/33) for the horizontal jump skill, 93.9% (n = 31/33) for the hop skill, and 93.9% (n = 31/33) for the one-leg balance skill. At 2-month follow-up, the completion rates for children’s FMS were: 93.1% (n = 27/29) for all five FMS, 96.6% (n = 28/29) for the overhand throw skill, 100% (n = 29/29) for the underhand throw skill, 96.6% (n = 28/29) for the horizontal jump skill, 100% (n = 29/29) for the hop skill, and 100% (n = 29/29) for the one-leg balance skill. For FMS that children did not complete, 0 points or seconds were awarded according to the pre-established criteria [27,38]. The reasons for incomplete FMS were that the children did not want to or struggled to perform the assessment or specific skill.

The primary and secondary raters scored 21.2% (n = 7/33) of the same videos at baseline and 20.7% (n = 6/29) of the same videos at 2-month follow-up to establish inter-rater reliability. The intraclass correlation coefficients (ICCs) for the overhand throw (ICC = 0.90, 95% CI = 0.72 to 0.97); underhand throw (ICC = 0.97, 95% CI = 0.90 to 0.99); hop (ICC = 0.90, 95% CI = 0.70 to 0.97); and one-leg balance (ICC = 0.99, 95% CI = 0.995 to 0.999) skill scores were greater than or equal to 0.90, except for the horizontal jump skill score (ICC = 0.79, 95% CI = 0.45 to 0.93).

### 3.3. Children’s FMS (Secondary Objective 1)

The final analytic sample was composed of 29 children. Before conducting paired samples *t*-tests, all data for FMS mean score differences were visually checked and assumed to be normally distributed. The results of paired samples *t*-tests for the mean changes in the children’s FMS scores between baseline and 2-month follow-up are presented in Table 4. There was a medium effect size for a positive change in the hop skill score from 1.31 (SD = 1.63) at baseline to 2.34 (SD = 2.48) at 2-month follow-up (*d* = 0.54). There were small effect sizes for mean changes in all other FMS scores (i.e., overhand throw, underhand throw, horizontal jump, and one-leg balance skill scores).

### 3.4. Parental Capability, Opportunity, and Motivation (Secondary Objective 2)

Table 5 represents the results of the repeated measures ANOVAs for the mean changes in parental capability, opportunity, and motivation between baseline, post-workshop, and 2-month follow-up. For parental capability, a large effect size was observed for a positive change in parental knowledge over time (partial *η*^2^ = 0.53). The positive change in parental knowledge found between baseline and post-workshop was maintained at 2-month follow-up. 

For parental opportunity, there was a large effect size for a positive change in the parental perceived availability of resources over time (partial *η*^2^ = 0.37). The positive change in the parental perceived availability of resources observed between baseline and post-workshop was maintained at 2-month follow-up. 

For parental motivation, there were large effect sizes for positive changes in parental confidence (partial *η*^2^ = 0.54) and parental beliefs (partial *η*^2^ = 0.26) over time. Additionally, a medium-to-large effect size for a positive change in parental outcome expectations was observed over time (partial *η*^2^ = 0.12). The positive changes found between baseline and post-workshop were sustained at 2-month follow-up for parental confidence and parental outcome expectations but not for parental beliefs.

### 3.5. Parental Physical Activity Modeling and Parent–Child Co-Participation in Physical Activity (Secondary Objective 3)

Table 5 shows the results of the repeated measures ANOVAs for the mean changes in parental physical activity modeling and parent–child co-participation in physical activity between baseline, post-workshop, and 2-month follow-up. There was a large effect size for a positive change in parental physical activity modeling over time (partial *η*^2^ = 0.20). In addition, a medium-to-large effect size for a positive change in parent–child co-participation in physical activity was observed over time (partial *η*^2^ = 0.13). The positive change in parental physical activity modeling found between baseline and post-workshop was maintained at 2-month follow-up. However, a positive change in parent–child co-participation in physical activity was found between baseline and 2-month follow-up but not between baseline and post-workshop.

## 4. Discussion

Building on previous in-person PLAYshop studies [23,24], our single-group mixed-methods pilot study was designed to explore the feasibility and some potential outcomes of the virtual PLAYshop program and subsequent assessment. To our knowledge, the current study is the first to explore the feasibility of a virtually delivered version of a parent-focused intervention aimed at enhancing parental capability, opportunity, and motivation to support their children’s physical literacy development. Additionally, prior to launching a full efficacy trial, we explored the virtual FMS assessment protocol for preschool-aged children, where the intention was to measure physical literacy more comprehensively within this age group in a future trial. Overall, the virtual PLAYshop program and the virtual assessment protocol for preschool-aged children’s FMS were feasible. The preliminary findings also suggest that the virtual PLAYshop program may improve parental capability, opportunity, and motivation to support their children’s physical literacy development and improve parental physical activity modeling and parent–child co-participation in physical activity. This parental behavior change may subsequently lead to improved physical literacy in their children. However, the efficacy of the virtual PLAYshop program in improving preschool-aged children’s FMS was unclear. Additionally, the efficacy in improving preschool-aged children’s physical literacy holistically has not yet been tested.

With respect to the feasibility of the virtual PLAYshop program, participants reported the high usefulness of, and satisfaction with, the virtual workshop. This finding aligned with the results of a previous PLAYshop study, wherein the high usefulness of, and satisfaction with, the original in-person PLAYshop workshop were also reported [23]. Overall, these findings suggest that both the virtual and in-person delivery of the PLAYshop program were acceptable to parents. A key advantage of the virtual format was that the PLAYshop program could be delivered safely during the COVID-19 pandemic. Given that COVID-19-related restrictions have unintentionally impacted FMS in preschool-aged children [49], it is important that programs, including virtual programs, exist. Additionally, the virtual PLAYshop program has the potential to provide benefits beyond the current COVID-19 pandemic. It could be used in future epidemics and pandemics. Moreover, it is possible that, outside of the pandemic, a hybrid intervention could be implemented that offers both virtual and in-person formats based on families’ preferences. However, one major advantage of the virtual format is that it can increase the scalability of the PLAYshop program in the future. More specifically, if the virtual PLAYshop program is found to be efficacious in a larger randomized controlled trial, the virtual format could expand the reach and potentially the benefits of the PLAYshop program with minimal cost implications. For example, it could extend the reach of the program into more rural and remote communities or to individuals with significant barriers (e.g., transportation). Regardless of the format for delivery, the brevity of a 75 min workshop, a core component of the PLAYshop program, is a great advantage in addressing parental time constraints when participating in purposeful play with their children [23,24]. It is also worth noting that this brevity was supplemented by other program components, providing educational and material resources and follow-up support after the workshop. However, some participants noted in the post-workshop questionnaire that the 75 min workshop was a bit long for keeping preschool-aged children engaged when the format was virtual. Therefore, future PLAYshop work should explore whether the virtual workshop can be condensed.

The qualitative findings regarding parental experiences of the virtual PLAYshop program were similar to the original in-person PLAYshop program [23,24], suggesting that parents benefited from the program, were highly satisfied, and were continuing activities that built their child’s physical literacy post-workshop. Parents also reported facilitators and barriers to their child’s participation in physical literacy activities post-workshop through qualitative interviews. The main facilitators and barriers were consistent between the virtual PLAYshop program and the in-person PLAYshop program [23,24]: facilitators of both included the simple nature of the activities, easy access to the activities, minimal equipment for the activities, and the child’s interest in the activities; barriers included a lack of parental time to engage in the activities, having siblings of varying developmental stages, limited indoor spaces, and unfavorable weather. Although limited indoor spaces and unfavorable weather emerged as barriers in both the virtual and in-person PLAYshop programs [24], some parents in the present study also mentioned that physical literacy activities from the virtual PLAYshop workshop would also be helpful indoors and on colder or rainy days.

Key to understanding the efficacy of the PLAYshop program is determining whether the program improved children’s physical literacy. It was not an objective of this pilot study to comprehensively assess children’s physical literacy; rather, the study focused on one aspect of physical literacy, where a virtual adaptation was required. Specifically, FMS are a key aspect of physical capability, a core component of physical literacy [11,50] that can be developed during early childhood [13]. Children with improved FMS tend to have increased physical activity [51], and children with increased physical activity tend to enjoy greater health benefits [1,2]. Though there are valid and reliable FMS assessment tools, such as the TGMD-3 [27,52], COVID-19 presented unique challenges for collecting in-person data on children. The virtual assessment protocol for preschool-aged children’s FMS piloted in the present study tried to balance the use of existing established tools and the logistical challenges of collecting data remotely and in homes with varying spaces and equipment. The findings of this study suggest that the virtual FMS assessment protocol is feasible to administer and score, although more training appears to be needed for the horizontal jump skill to enhance its reliability. It is important to note that physical capability (e.g., FMS) is only one component of physical literacy; therefore, future research examining the efficacy of the PLAYshop program should simultaneously assess other components of physical literacy (e.g., affective) where feasible in preschool-aged children in order to test the efficacy of the intervention in improving preschool-aged children’s physical literacy holistically [11,53].

In terms of our secondary objective, examining whether the PLAYshop program had an impact on preschool-aged children’s FMS, improvement was only observed in the hop skill score, though this improvement was characterized by a medium effect size. This was interesting as the manipulative/ball skills, which often receive less focus in formal early years settings [54], were an important focus of the virtual workshop activities. This may have reflected the developmental stages of the children. For example, proper preparatory positioning (windup) and rotating and stepping for the overhand throw typically appear at 5–6 years of age, and fully mature patterns appear at 7–8 years of age [55,56]. Similarly, in this study, all four items for assessing the overhand throw (i.e., proper preparatory positioning, rotating, stepping, throwing) showed low scores (data not shown). Since this could be an assessment timing issue rather than an intervention issue, these findings do not preclude the need to incorporate foundational activities that build preschool-aged children’s manipulation/ball skills into the intervention. Notably, our findings are in line with previous studies of in-person physical activity interventions delivered in childcare settings that have observed improvements in children’s locomotor skills but not manipulative/ball skills [57,58]. Additionally, while the qualitative data revealed that all participants had intentions of continuing physical literacy activities in the future, capturing the dose or how often parents and children actually engage in physical literacy activities post-workshop should be incorporated into future PLAYshop trials to aid in the interpretation of findings regarding FMS. Finally, although completion rates for the balance skill assessment were high (baseline: 93.9%; 2-month follow-up: 100%), some children lost motivation to balance as long as possible on this skill assessment, showing low average times (baseline: 5.23 s; 2-month follow-up: 6.16 s). Accordingly, it is recommended to find ways to increase preschool-aged children’s motivation to perform the balance skill as long as possible. Making the FMS assessment, especially the balance skill, more game-like [59] and using a different balance skill assessment tool, where a maximum balance time is incorporated (e.g., the balance test of the Movement Assessment Battery for Children-Second Edition (MABC-2) [60]), might address this issue.

The preliminary findings indicated that the virtual PLAYshop program has the potential to be efficacious in achieving parental outcomes. Of note, large effect sizes for improvements in parental capability (i.e., knowledge); opportunity (i.e., perceived availability of resources); motivation (i.e., confidence, beliefs); and physical activity modeling were observed over time. Additionally, medium-to-large effect sizes for improvements in parental motivation (i.e., outcome expectations) and parent–child co-participation in physical activity were observed over time. Previous in-person PLAYshop studies captured these indicators at only two time points, baseline and post-workshop [23,24]. However, this study uniquely captured these indicators at three time points, including at 2-month follow-up, to determine whether mean changes found between baseline and post-workshop were maintained at 2-month follow-up. For example, positive changes in parental knowledge, the perceived availability of resources, and confidence were observed in both the virtual and in-person PLAYshop studies [24], but this virtual PLAYshop study confirmed that these positive changes were maintained at 2-month follow-up. It is noteworthy that positive changes in parental beliefs and outcome expectations were not found in the in-person PLAYshop study [24], but these positive changes were observed in the present study, and the positive change in parental outcome expectations was maintained at 2-month follow-up. Given the emphasis placed on the role of parents in developing their children’s physical literacy [13], the findings across the virtual and in-person PLAYshop studies [23,24] are promising. The findings from our virtual PLAYshop study need to be tested with a higher-quality design and larger sample to evaluate its efficacy.

This study had several notable strengths. First, quantitative findings were provided alongside qualitative process evaluation findings to explore parental implementation and experiences of the virtual PLAYshop program across multiple dimensions. Second, preschool-aged children’s FMS were directly observed through the development of a virtual FMS assessment protocol. Finally, the virtual PLAYshop program was designed to build parental capability, opportunity, and motivation to support their preschool-aged children’s physical literacy development, particularly through purposeful play. Growing bodies of research and practice demonstrate that the active learning approach, which can be achieved through purposeful play, offers greater cognitive and social-emotional benefits to children over traditional passive learning [61]. Regardless, this study also had limitations. Although the pilot study was a valuable step toward a larger randomized controlled efficacy trial [28], there was no control group, and the number of participants was small (baseline n = 34); thus, the findings regarding the secondary objectives should be interpreted with caution. Finally, due to the self-reported nature of the parental online questionnaire, the influence of social desirability bias on parental outcomes could not be ruled out. However, the findings of the current study as well as previous studies [24,39,40] suggest that the parental measures were reliable.

## 5. Conclusions

This single-group pilot study provided an initial evaluation of a virtually delivered version of a theory-based, parent-focused physical literacy intervention for early childhood (the PLAYshop program). The virtual PLAYshop program and a virtual assessment protocol for preschool-aged children’s FMS were found to be feasible. This program may be efficacious in improving parental capability, opportunity, and motivation to support their children’s physical literacy development, as well as parental physical activity modeling and parent–child co-participation in physical activity. However, its impact on preschool-aged children’s physical literacy was unclear. Given the feasibility and potential positive outcomes of the virtual PLAYshop program observed in this study, the next step is to conduct a larger randomized controlled trial with a control group and a more comprehensive assessment of children’s physical literacy to evaluate the efficacy of this program. The findings and limitations identified in this pilot study provide critical information for scaling up to definitive and more extensive efficacy studies.

## Figures and Tables

**Table 1:** Descriptive characteristics of participating children and parents.

Participant Characteristics	Parent Online Questionnaire			Virtual Assessment Session for Children’s FMS ^b^	
	Baseline (n = 34)	Post-Workshop (n = 32)	2-Month Follow-Up (n = 30)	Baseline (n = 33)	2-Month Follow-Up (n = 29)
**Children’s characteristics**					
**Age** (**years**)	4.37 (0.13)	4.37 (0.14)	4.31 (0.14)	4.36 (0.13)	4.30 (0.14)
**Sex** (**assigned at birth**)					
Male	55.9 (19)	56.3 (18)	56.7 (17)	54.5 (18)	55.2 (16)
Female	44.1 (15)	43.8 (14)	43.3 (13)	45.5 (15)	44.8 (13)
**Race/ethnicity**					
White	73.5 (25)	75.0 (24)	73.3 (22)	75.8 (25)	75.9 (22)
Other	26.5 (9)	25.0 (8)	26.7 (8)	24.2 (8)	24.1 (7)
**Number of children**					
1	23.5 (8)	21.9 (7)	23.3 (7)	24.2 (8)	24.1 (7)
2	38.2 (13)	40.6 (13)	40.0 (12)	39.4 (13)	41.4 (12)
3 or more	38.3 (13)	37.6 (12)	36.6 (11)	36.4 (12)	34.4 (10)
**Nonparental care time** (**hours per week**)	17.93 (3.03)	16.23 (2.96)	16.15 (3.05)	17.56 (3.10)	15.67 (3.11)
**Full-time care ^a^**					
Yes	38.2 (13)	34.4 (11)	33.3 (10)	36.4 (12)	31.0 (9)
No	61.8 (21)	65.6 (21)	66.7 (20)	63.6 (21)	69.0 (20)
**Parental characteristics**					
**Age** (**years**)	37.56 (0.74)	37.62 (0.75)	37.78 (0.78)	37.59 (0.76)	37.82 (0.81)
**Sex** (**assigned at birth**)					
Male	5.9 (2)	6.3 (2)	6.7 (2)	6.1 (2)	6.9 (2)
Female	94.1 (32)	93.8 (30)	93.3 (28)	93.9 (31)	93.1 (27)
**Highest level of education**					
Above Bachelor’s degree	29.4 (10)	31.3 (10)	30.0 (9)	30.3 (10)	31.0 (9)
Bachelor’s degree	52.9 (18)	50.0 (16)	53.3 (16)	51.5 (17)	51.7 (15)
Below Bachelor’s degree	17.6 (6)	18.8 (6)	16.7 (5)	18.2 (6)	17.2 (5)
**Previous physical literacy-related training**					
Yes	26.5 (9)	28.1 (9)	26.7 (8)	24.2 (8)	24.1 (7)
No	73.5 (25)	71.9 (23)	73.3 (22)	75.8 (25)	75.9 (22)

**Note:** Values show mean (standard error) for continuous values (children’s age, nonparental care time, and parental age) and percentage (frequency) for categorical values (children’s sex, children’s race/ethnicity, number of children, full-time care, parental sex, parental highest level of education, and parental previous physical literacy-related training). The mean (standard error) is presented to two decimal places and the percentage to one decimal place. ^a^ Full-time care was defined as nonparental care time for 30 h or more per week [42]. ^b^ Fundamental movement skills (FMS).

**Table 2:** Physical literacy activities performed since the virtual workshop, with sample quotes.

Physical Literacy Activities	Sample Quotes
Manipulative/ball skill activities	PS04: We’ve done lots of games with balls and like we have a little soccer net, so just kicking the ball around to each other and to the soccer net.
	PS18: He’s really drawn to the paddle with the balloons… he got his older brother and older sister in on it, so they were playing kind of ‘keepy uppy’ and wapping it against the wall.
	PS32: And he obviously loves this blue ball. He’s always like throwing and catching and sometimes we’ll play catch.
Locomotor skill activities	PS21: We’ve been like when we go on walks, we try to do shadow tag, and try to do two foot jumps over things and make sure we’re landing with both feet.
	PS30: We did the one where you cut out shapes and do like the hopscotch with the colors.
Balance skill activities	PS03: She’s actually been practicing the, the one legged balance thing. I think it’s on one of her yoga sheets as well, it’s the same thing. So she’s been practicing that often. She’s super proud of how well she can balance now.
	PS23: She balances on just like fencing and logs that are knocked over or like balance and jump from like stone to stone and like walk along the logs.
Other activities	PS15: Riding bikes, or jumping on the trampolines is probably his favorite.
	PS19: And then mostly we’ve been focusing on bike riding and swimming this summer, so we’ve been doing lots of that.
Parent–child co-play	PS04: Daddy pretends he’s a sleeping alligator and they sneak up and then he tries to catch them and they run away.
	PS32: Now I think I understand that it is important for both of us to engage in these activities. And like you said, to make it fun, you don’t want it to be something that you dread doing right. Make enjoyable for the both of you.

**Table 3:** Facilitators and barriers, with sample quotes.

Facilitators and Barriers	Sample Quotes
Parent	
Physical literacy knowledge *	PS10: I definitely have a better idea of how to get him moving. And you made it easier. (facilitator)
	PS16: It was funny because it wasn’t until the workshop that I realized there were a couple of things that he had never done… like an underhanded throw was one. (facilitator)
Personal factor: parental energy	PS14: Our energy levels as adults to sort of facilitate these is not always there depending on the day. (barrier)
	PS25: I am working casually now, so I think that’s a big, that’s a big one because I’m not tired as much. I can schedule the time. (facilitator)
Personal factor: making it a habit/adding it to routine	PS07: That’s one piece, like prioritizing the time to really practice that active play without signing up for a class or, um, you know, something like that. I would say it’s something that I feel a little bit sad about because there’s so much more that we know now that we can be doing. (barrier)
	PS23: What if I can see a little opportunity here or there, like chasing each other around after bath, to do that and let her run around and jump on the couches and throw stuff in the house within reason. (facilitator)
Personal factor: caring for other children	PS27: I have a younger (child) that’s eight months. So when he started crawling and moving around, the combo of the two not being the same age, finding common activities was tricky. (barrier)
Cost of activities	PS04: This was a nice reminder of things that we can be doing that are simple at home and not like on a huge scale that, you know, they have to be registered in a $200 a day program of doing skills of soccer or whatever. (facilitator)
Child	
Personal factor: child’s energy	PS08: He’s at daycare all day and when he gets home, he’s kind of done. They do over two hours of outside play at daycare each day. It’s go, go, go all day. So when he gets home he kind of wants to veg it seems. (barrier)
	PS34: She’s very active, she likes to play, she likes to be outside. She likes to play with her baby sister. She’s an active girl. She has lots of energy. (facilitator)
Personal factor: child’s mood	PS20: I would say that if he’s in a mood and he wants to play, they go really well. If he’s in a mood where that’s not his idea, then they don’t go well at all. (facilitator and barrier)
Personal factor: child’s interest	PS07: She was already interested in throwing, but the workshop really gave us some more pieces to think about, like how you throw. (facilitator)
Playful experiences *	PS07: She also really loves putting pillows on the floor and figuring out how to get from one place to another. (facilitator)
	PS08: Sometimes he has his own ideas of how things should go, so I might think, oh, we’re going to do such and such. And then he’s like, no mom, we’re going to do it this way. And because it’s play, I usually just let him go with his ideas and stuff. So yeah, he’s not always that coachable, but it’s gone pretty well. (facilitator)
	PS18: This feels a little bit more like we’re having fun, we’re playing a game, but I’m more confident about what the objective is with it, you know, their objective is always have fun. (facilitator)
Environment	
Climate	PS10: The summer is always nice too, just to be able to get outside and run in the sprinkler and play with the balls and stuff like that. So summer is always great. (facilitator)
	PS14: And I think sort of indoor activities, you know, it’s summer right now, it’s pretty easy for us to be outside and be more active. But sometimes I’m looking forward to trying them in the winter or on days where it’s not quite so nice, right. Where it’s a bit tougher sometimes. (facilitator and barrier)
	PS20: As much as it probably shouldn’t affect things, I would say the weather makes a big difference. (barrier)
Location	PS03: Our living room is very much like a play area anyway… so there’s just lots of room to play. (facilitator)
	PS15: We have a fairly decent sized backyard and a park close by, so we can go to either of those places to do it. (facilitator)
	PS16: Honestly, that was probably the most helpful thing is all those ideas that I hadn’t thought of that will work so great inside. (facilitator)
	PS32: I just wish we had a little bit more space. Living in a condo is we’re very restricted in terms of space. (barrier)
Minimal equipment *	PS14: Pointing out where we could use household items was helpful. (facilitator)
	PS14: The setup’s pretty easy. (facilitator)
	PS19: It’s really easy to have something super fun and engaging that doesn’t need a lot of big fancy equipment. (facilitator)
Finding time	PS01: Just busyness, once the nights start getting dark earlier, it feels like we run out of time once we’re home from school and work to go out and do something. (barrier)
	PS04: I think that that was important that they were simple and could be easily done, because if you had given us ideas, like, okay, you need to buy this equipment and you need to do this and this, and it’s going to take you an hour each day. It would be just too overwhelming (facilitator)
	PS18: We don’t need something fancy or preplanned and meticulously organized… I feel more like they’re spontaneous now, but they’re purposeful. (facilitator)
COVID-19 pandemic	PS14: I think the other thing, I guess I’ll mention is this is an interesting time to do it where COVID has interrupted a lot of these like registered activities. (facilitator)
	PS25: COVID restrictions are down a little bit, so that’s been also something like, I don’t feel guilty much that I’m not spending too much time, because then he can do those activities in his camps and with his team. (barrier)

* No quotes by parents supported this theme as a barrier.

**Table 4:** Results from paired- samples *t*-tests for the mean changes in virtually assessed children’s FMS scores between baseline and 2-month follow-up (n = 29).

Variables (Score Range)	Baseline		2-Month Follow-Up		Effect Size	*p*	95% CI	
	M	SD	M	SD	(Cohen’s *d*)		Lower	Upper
**Children’s FMS**								
Overhand throw (0–8)	1.00	1.65	0.90	1.42	−0.06	0.742	−0.74	0.53
Underhand throw (0–8)	3.59	3.01	3.59	2.60	0.00	1.000	−1.23	1.23
Horizontal jump (0–8)	2.00	1.56	2.10	1.54	0.07	0.729	−0.50	0.71
Hop (0–8)	1.31	1.63	2.34	2.48	0.54	0.007	0.31	1.76
One-leg balance ^a^	5.23	9.02	6.16	10.24	0.24	0.204	−0.53	2.39

**Note:** M = mean; SD = standard deviation. ^a^ Seconds (no range).

**Table 5:** Results from repeated measures ANOVAs for the mean changes in parental capability, opportunity, motivation, physical activity modeling, and parent–child co-participation in physical activity between baseline, post-workshop, and 2-month follow-up (n = 30).

Parental Self-Reported Outcome Variables (Score Range)	Baseline (T1)		Post-Workshop (T2)		2-Month Follow-Up (T3)		Effect Size	*p*	Within-Subjects Contrasts
	M	SE	M	SE	M	SE	(Partial *η*^2^)		
**Parental capability**									
Parental knowledge (0–45)	27.29	1.32	33.26	0.98	34.27	0.82	0.53	<0.001	T1 < T2; T1 < T3
**Parental opportunity**									
Parental perceived availability of resources (0–5)	3.43	0.14	4.13	0.13	4.27	0.14	0.37	<0.001	T1 < T2; T1 < T3
Parental perceived barrier: lack of time (0–5)	2.10	0.14	1.73	0.13	2.10	0.19	0.06	0.178 ^a^	
Parental perceived barrier: discomfort in letting child play outside (0–5)	2.07	0.15	1.87	0.15	2.17	0.15	0.05	0.266	
Parental perceived barrier: lack of opportunity (0–5)	2.13	0.17	1.93	0.15	2.10	0.15	0.02	0.552	
Parental perceived barrier: transportation problem (0–5)	2.00	0.16	1.87	0.14	2.00	0.14	0.02	0.594	
Parental perceived barrier: high cost (0–5)	3.10	0.22	3.13	0.22	3.13	0.22	0.00	0.968	
**Parental motivation**									
Parental confidence (0–55)	36.61	1.42	44.37	1.18	44.78	1.00	0.54	<0.001	T1 < T2; T1 < T3
Parental beliefs (0–20)	17.24	0.35	18.53	0.30	17.67	0.39	0.26	<0.001	T1 < T2
Parental outcome expectations (0–15)	12.60	0.26	13.15	0.28	13.14	0.23	0.12	0.027	T1 < T2; T1 < T3
**Other**									
Parental physical activity modeling (0–12)	7.93	0.34	8.70	0.34	8.87	0.33	0.20	0.001	T1 < T2; T1 < T3
Parent–child co-participation in physical activity (0–20)	13.57	0.68	14.12	0.59	14.97	0.52	0.13	0.028 ^a^	T1 < T3

**Note:** M = estimated marginal mean; SE = standard error. ^a^ Greenhouse–Geisser.

## Data Availability

Data and materials are available upon reasonable request.

## Supplementary Materials

The following supporting information can be downloaded at: https://www.mdpi.com/article/10.3390/children10040720/s1, Supplementary Table S1: Results of missing values analyses.
