# Supplementary material for: Piloting the Virtual PLAYshop Program: A Parent-Focused Physical Literacy Intervention for Early Childhood
Source: Children (Basel). 2023 Apr 13;10(4):720. doi: 10.3390/children10040720 (PMC10136860; doi:10.3390/children10040720)
Supplement: Supplementary file 1 [file children-10-00720-s001.zip › children-2269869-supplementary.pdf]

Supplementary file 1. Results of missing values analyses

Variables at all time points (baseline, post-workshop, 2-month follow-up)	Results of Little's missing completely at random test	Overall summary of missing values	
		Cases % (n)	Values % (n)
**All variables**	*χ^2=8.660, df=1,092, p=1.000*	30.00 (9)	0.33 (15)
**Parental capability**			
Parental knowledge	*χ^2=56.958, df=51, p=0.263*	6.67 (2)	0.37 (3)
**Parental opportunity**			
Parental perceived availability of resources	*no missing values*		
Parental perceived barriers	*χ^2=16.677, df=14, p=0.274*	3.33 (1)	0.22 (1)
**Parental motivation**			
Parental confidence	*χ^2=108.259, df=127, p=0.884*	13.33 (4)	0.51 (5)
Parental beliefs	*χ^2=16.044, df=11, p=0.140*	3.33 (1)	0.28 (1)
Parental outcome expectations	*χ^2=4.148, df=16, p=0.999*	6.67 (2)	0.74 (2)
**Other**			
Parental physical activity modeling	*χ^2=3.021, df=8, p=0.933*	3.33 (1)	0.37 (1)
Parent-child co-participation in physical activity	*χ^2=30.848, df=22, p=0.099*	6.67 (2)	0.56 (2)
